# Fluctuation in systolic blood pressure is a major systemic risk factor for development of primary open-angle glaucoma

**DOI:** 10.1038/srep43734

**Published:** 2017-03-06

**Authors:** Na Young Lee, Younhea Jung, Kyungdo Han, Chan Kee Park

**Affiliations:** 1 Department of Ophthalmology and Visual Science, Incheon St. Mary’s Hospital, College of Medicine, The Catholic University of Korea, Seoul, Korea; 2 Department of Ophthalmology and Visual Science, Seoul St. Mary’s Hospital, College of Medicine, The Catholic University of Korea, Seoul, Korea; 3 Department of Biostatistics, The Catholic University of Korea, Seoul, Korea

## Abstract

We evaluated the risk of development of primary open-angle glaucoma (POAG) in terms of variability in BP using a nationwide, population-based, 11-year longitudinal study using the Korean National Health Insurance Research Database. We included patients who underwent health care examinations more than twice between January 2002 and December 2006. We divided all subjects by the quartiles of variability in systolic blood pressure (SBP), diastolic blood pressure (DBP), and the difference between SBP and DBP. Of the total of 80,021 included subjects, 910 were diagnosed with POAG between January 2007 and December 2013. Both the Kaplan-Meier survival curves and log-rank test data indicated that patients with higher-level BP variability developed POAG significantly more frequently than did patients with lower-level variability (P < 0.001). On multivariate Cox’s regression modeling including gender, age, sex, household income, smoking status, level of alcohol intake, extent of exercise, diabetes mellitus status, dyslipidemia status, SBP, and DBP; the hazard ratios among the highest and lowest quartiles of SD SBP and CV SBP were 1.256 and 1.238, respectively. Our findings suggest that subjects in the highest quartile of SBP variability were significantly more likely to develop POAG in our population-based sample of Korean adults.

Glaucoma is commonly defined as a progressive optic neuropathy accompanied by characteristic structural damage to the optic nerve, and visual field loss^1^,^2^. Risk factors for glaucoma development include elevated intraocular pressure (IOP), age, a family history, the clinical appearance of the optic nerve, race, thinner central corneal thickness and the potential for vascular disease^3^,^4^,^5^,^6^,^7^.

The suggested pathological cause of primary open angle glaucoma (POAG) is elevated IOP, and IOP control is the only proven effective treatment^8^,^9^. Several large, randomized clinical trials have revealed a relationship between IOP and glaucoma development and progression^8^,^9^,^10^,^11^,^12^. Apart from the mechanical effects of an elevated IOP on the optic nerve head, the peripapillary connective tissue and the ganglion cells, several vascular factors have also been identified as risk factors^3^,^12^,^13^,^14^,^15^,^16^. An increase of systolic blood pressure (BP) and diastolic BP is related to a higher mean IOP^17^ and hypertension is regarded as a systemic risk factor for POAG development in several studies^6^,^18^,^19^. Systemic hypertension may trigger an increase in IOP induced via overproduction of aqueous humor or impaired outflow of humor from the eye^20^. However, the mechanism by which elevated BP causes glaucoma remains poorly understood and, indeed, the relationship between glaucoma and BP remains a topic of debate.

In 2010, Rothwell *et al*. were the first to reliably investigate the association between non-situational, within-individual visit-to-visit variability in BP and the subsequent risk of cardiovascular events, and the cited authors concluded that high-level BP variability was harmful in patients with established vascular disease^21^. Such variability was independent of both the mean BP and other traditional cardiovascular risk factors^21^,^22^,^23^.

BP variability may trigger ischemic stroke because long-term repetitive peaks and troughs in BP may damage small cerebral vessels, possibly by inducing endothelial dysfunction and breakdown of the blood–brain barrier^24^. The vascular theory of glaucoma pathogenesis proposes that a vascular abnormality including ischemic injury and abnormalities in vascular autoregulation is the underlying cause of the optic atrophy and ischemia/reperfusion injury triggers glaucomatous optic neuropathy^25^,^26^,^27^. Similarly, we hypothesized that patients exhibiting higher-level BP variability might suffer a higher incidence of POAG.

To the best of our knowledge, no population-based cohort study has yet explored the association between BP variability and POAG development. We addressed this question using a nationally representative sample of 1,025,340 South Korean adults of the National Health Insurance Service National Sample Cohort 2002–2013 (NHIS-NSC 2002–2013).

## Methods

### Study population

The Korean National Health Insurance Service (KNHIS) constructed the NHI database between 2002 and 2013, which is a sample of the entire South Korean population of all ages (total 46,605,433). The data are linked to information on socio-demographic variables, mortality, medical service utilization, and health examination status using unique personal identification numbers. The 2002 data are from 1,025,340 nationally representative, randomly chosen subjects, constituting approximately 2.2% of the entire KHNIS population. We used NHIS-NSC 2002–2013 data, which were released by the KNHIS in 2015. The database includes information on all medical claims filed from January 2002 to December 2013. No healthcare records were duplicated or omitted because all Korean residents receive a unique identification number at birth. Individual data were linked to medical claim data provided by Statistics Korea using the unique 13-digit personal identification numbers.

The NHIS-NSC 2002–2013 project was approved by the Institutional Review Board of the KNHIS. Our study design was reviewed and approved by the Institutional Review Board of Incheon St. Mary’s Hospital, South Korea. We followed all tenets of the Declaration of Helsinki. The need for written informed consent was waived by our Review Board.

The KHNIS covers approximately 97% of the Korean population and is a compulsory form of social insurance. The system pays some POAG-related healthcare costs. Patients pay 30% of their total medical expenses, and medical providers submit claims for the remaining 70%. Claims are accompanied by information on diagnosis, procedures performed, prescriptions issued, demographic data, and direct medical costs. The diagnostic codes used by the KNHIS are based on the International Classification of Disease, 10^th^ revision (ICD-10). All prescription medicines for glaucoma are covered by the KHNIS. This means that the combination of a relevant diagnostic code and prescription of glaucoma medicine serves to definitively diagnose POAG. We identified POAG cases registered from 2002 through 2013 by reference to the initial POAG diagnostic codes (ICD-10 codes ‘H401’, ‘H408’, and ‘H409’) and prescription of antiglaucoma medications. We included patients over the age of 40 years, but excluded subjects diagnosed with POAG to December 2006 (n = 440). This ensured that the POAG group included only patients with newly developed disease.

We included subjects for whom more than two BP measurements, taken between January 2002 and December 2006, were available. In the KNHIS, the equipment used to BP measurements varies between sites. However, most people performed their medical examinations at the same hospital near their residence, most BP measurements were undertaken using the same equipment in each individual. BP was measured by trained clinicians. Systolic and diastolic BP were measured separately, and the sitting brachial BP was the average of two measurements taken after the subject had been seated for 5 min with an arm in the appropriate position. We used the mean SBPs and DBPs measured at each visit to calculate the standard deviations (SDs) in SBP and DBP over the various visits. The difference in BP was the SBP minus the DBP; we also calculated the SD of this BP difference over the course of clinical visits^21^. Visit-to-visit BP variability is defined using these SDs, or coefficients of variation (CVs; a CV is the SD/mean).

Detailed histories of smoking status, alcohol consumption, and physical activity (including the amount and frequency of such activity) were obtained via questionnaire. To permit statistical analysis, we simplified smoking status into current, former, or never; alcohol consumption into ≤three times/month, ≥once/week, or none; and physical activity into no activity, ≤four times/week, or ≥five times/week. Household income and residential area were categorized, respectively, into the lowest 20% and the highest 80% of income level, and as urban or not. Comorbidities were identified using information gathered in the 1 year prior to the index date and included diabetes mellitus (ICD-10 codes E11-E14), hypertension (ICD-10 codes I10, I11, I12, I13, and I15), and dyslipidemia (ICD-10 code E78).

### Statistical Analysis

We report the means ± SDs, with intervals, of continuous variables, and the numbers (with percentages) of categorical variables. Baseline characteristics were compared among the POAG and other groups using the chi-squared and t-tests.

To identify the risk of POAG by the quartile of BP variability, we calculated hazard ratios (HRs) with 95% confidence intervals (CIs) and analyzed these data using a Cox’s proportional hazard regression model. All subjects were divided into four quartiles, Q1–Q4, in terms of the SD or CV of SBP, DBP, and the difference between SBP and DBP. The HRs for POAG associated with SD quartiles, or the CVs of SBP, DBP, and the BP difference, were calculated; the lowest quartile served as the referent. We analyzed associations between BP variability and POAG development using four models. Model 1 included BP variability, age, and sex. Model 2 included other confounders: smoking status, extent of physical activity, alcohol intake, and household income. Model 3 additionally included diabetes mellitus and dyslipidemia status. Finally, Model 4 additionally included SBP and DBP.

The cumulative POAG incidence was estimated by constructing Kaplan–Meier curves for the entire 7-year follow-up period, and we used the log-rank test to examine differences in POAG development by the quartile of BP variability. A *P* value < 0.05 was considered to reflect statistical significance. SAS version 9.3 software, and SAS survey procedures (SAS Institute, Inc., Cary, NC, USA), were used for all statistical analyses.

## Results

**Figure 1:**
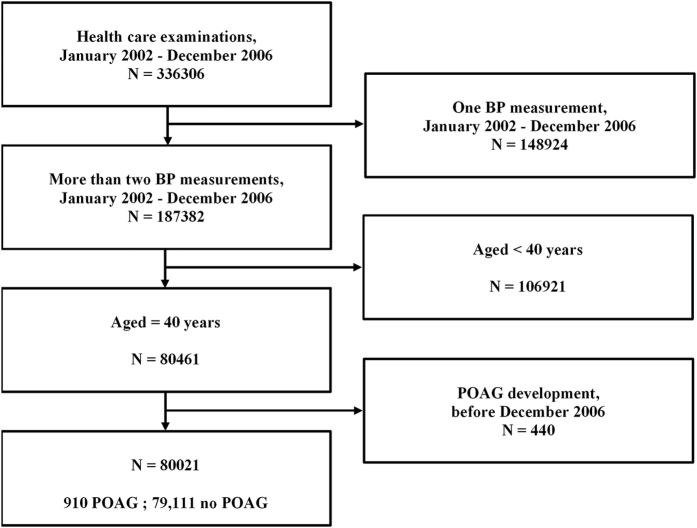
Flow chart of the study population. POAG = primary open-angle glaucoma.

Figure 1 shows a workflow chart. We identified 910 POAG patients in our cohort; 79,111 subjects did not have POAG. The average BP measurements were 3.07 and median number of BP measurements was 3 times. Table 1 shows the characteristics of the two cohorts and, thus, the POAG and comparison groups. POAG patients were more likely to be older (P < 0.0001), non-smokers (P = 0.0141), non-drinkers (P = 0.0365), and to take more exercise (P = 0.0126), than subjects of the comparison group. However, we found no significant between-group difference in terms of any of smoking status, alcohol consumption, or physical activity, after adjustment for age. The frequencies of diabetes mellitus (P < 0.0001), hypertension (P < 0.0001), and dyslipidemia (P < 0.0001) differed significantly between the groups both before and after age-adjustment. No significant difference in any of sex, household income, or residential area was evident between the two groups. POAG patients were more likely to have a higher SBP (P < 0.0001) and DBP (P = 0.0029) than were subjects of the comparison group, but the difference in DBP was not significant after age-adjustment.

Visit-to-visit variability in SBP and DBP (defined using SDs or CVs) differed significantly both before and after age-adjustment between the two groups. The SD of the BP difference differed significantly before age-adjustment. However, neither the SD of the BP difference after age-adjustment, nor the CVs of the BP differences before and after age-adjustment, differed significantly between the two groups.

Table 2 shows the results of Cox’s proportional hazard regression modeling by the quartiles of the SDs or CVs of SBP, DBP, and the BP difference. After adjusting for age, sex, household income, alcohol intake, extent of exercise, diabetes mellitus, and dyslipidemia (Model 3), multivariate Cox’s regression analysis showed that subjects in the fourth quartiles of SBP SD or CV were significantly more likely to develop POAG (HRs, 1.261; 95% CI, 1.036–1.536; 1.233; 95% CI, 1.012–1.502, respectively). Upon further adjustment by addition of SBP and DBP (Model 4), these associations remained (HRs, 1.256; 95% CI, 1.030–1.531; 1.238; 95% CI, 1.016–1.508, respectively). DBP variability also tended to be associated with POAG development, but was less predictive than was SBP variability. Subjects in the third DBP SD quartiles were significantly more likely to develop POAG when Models 2, 3, and 4 were run. However, no DBP CV quartile was significantly associated with such development. On Cox’s proportional hazard regression, we found no significant association between POAG development by SD or CV quartile of the BP difference.

**Figure 2:**
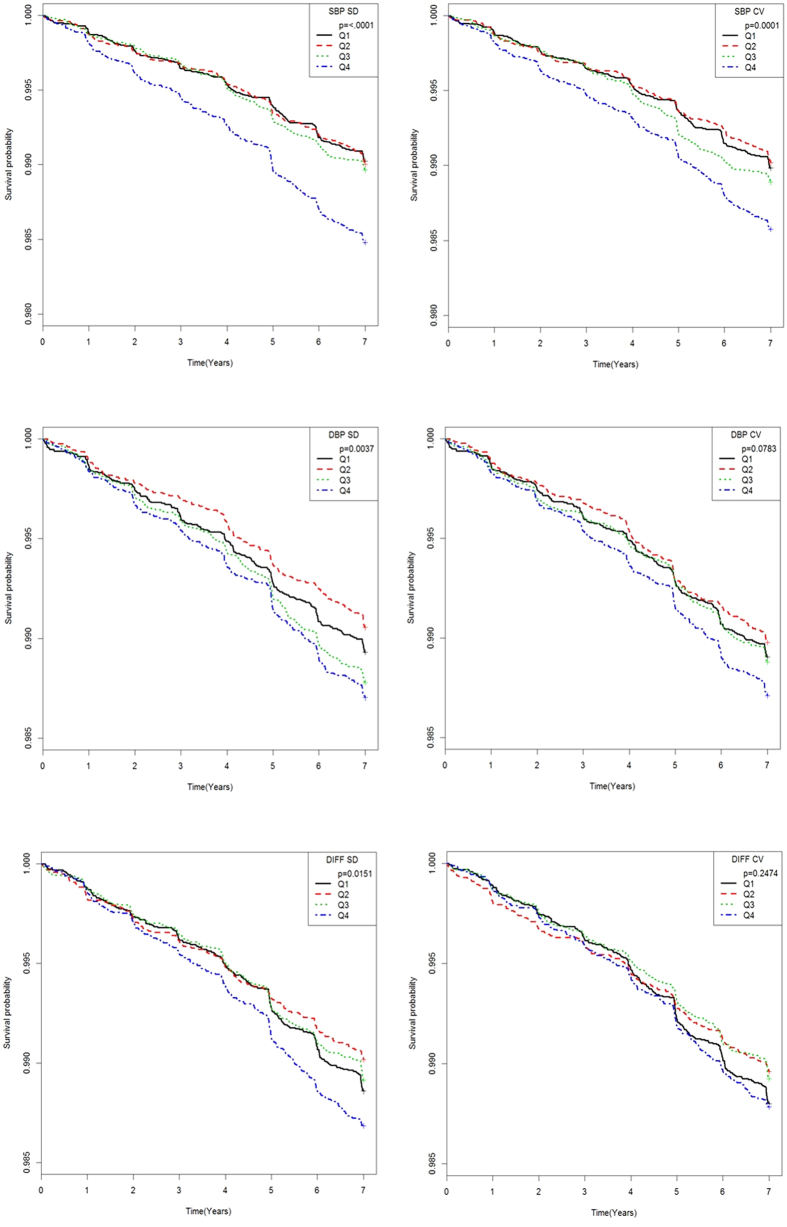
Kaplan-Meier survival curves of cumulative incidence of primary open-angle glaucoma (POAG) in patients according to quartiles (Qs) of the SD or CV of systolic blood pressure (SBP), diastolic blood pressure (DBP) and difference between SBP and DBP. Patients with the highest SD or CV of SBP (Q4) had significantly higher cumulative development of POAG than did those in the other quartiles (P < 0.0001 by log-rank test).

Figure 2 showed the results of Kaplan-Meier survival curves and log-rank testing. The latter data indicated that patients in quartile 4 of SBP variability developed POAG considerably more frequently than did patients with lower-level BP variability; statistical significance was attained (P < 0.0001).

Table 3 lists the characteristics of the study population by SBP SD quartile. We divided the study population into two groups (quartiles [Q]1, Q2 and Q3 compared with Q4) to evaluate further the characteristics of patients exhibiting high-level SBP variability. Those in the fourth SBP SD quartile were more likely to be older (P < 0.0001), female (P < 0.0001), non-smokers (P < 0.0001), non-drinkers (P < 0.0001), and to take less exercise (P < 0.0001), than those of other quartiles. The frequencies of diabetes mellitus, hypertension, and dyslipidemia also differed significantly between Q4 patients and those of other quartiles (P < 0.0001 for all comparisons). Q4 subjects were more likely to have a higher DBP, and higher glucose and total cholesterol levels, than were those of the other three quartiles (P < 0.0001 for all comparisons).

## Discussion

To the best of our knowledge, this is the first study to show that SBP variability is associated with POAG development, independently of SBP, in a large population-based cohort. BP variability is commonly defined as variability in BP over time; thus, the SD or CV of such variability^21^,^28^. BP variability plays important roles in the progression of organ damage, triggering various vascular events^28^. Visit-to-visit SBP variability is a strong predictor of stroke, independent of the mean SBP^16^. In addition, the microangiopathy of hypertension can cause end-organ damage to, for example, the retina and optic nerve^29^. Risk of ischemia is likely to be greatest if both variability and instability are increased such that changes in blood pressure are sudden and large^28^.

Although several large epidemiological studies have investigated the relationship between POAG and BP, the data are conflicting^30^. Several studies found that individuals with elevated BP were at a low risk of glaucoma^31^,^32^,^33^,^34^, whereas other cross-sectional works reported significant associations between high-level systemic BP and POAG^17^,^18^,^35^,^36^. Plange *et al*. investigated the difference of BP fluctuation between glaucoma patients and controls using 24-h BP monitoring and Lee *et al*. evaluated relationship between daytime variability of BP or ocular perfusion pressure (OPP) and visual field progression^37^,^38^. However, no population-based cohort study has yet evaluated the relationship between POAG and BP variability

The complex relationship between BP and POAG development may reflect the fact that many factors contribute to glaucoma development. An elevated IOP is an important cause of glaucomatous optic neuropathy. In addition, vascular risk factors, including disc hemorrhage, vascular and systemic diseases, and migraine, play important roles^39^,^40^. Of these risk factors, BP is thought to be important and should be consider both BP itself and OPP calculated as BP minus IOP^32^. In the Rotterdam Study, hypertension defined as a SBP of 160 mmHg or higher and/or a DBP of 95 mmHg or higher was associated with high-tension glaucoma but not normal-tension glaucoma^17^. In the Thessaloniki Eye Study, a low diastolic OPP was associated with an increased risk of POAG in patients on antihypertensive treatment^32^. In addition to these previous studies, we have shown that visit-to-visit SBP variability is a powerful predictor of POAG, independently of the mean SBP.

The clinical significance of visit-to-visit SBP variability in POAG patients may be explained by reference to the pathophysiological mechanism of glaucoma. Glaucomatous optic neuropathy is characterized by the eventual death of retinal ganglion cells. Such death occurs via apoptosis caused by disturbance to the retinal blood flow, ischemia-reperfusion injury, metabolic derangement, with reactive oxygen species overload, the synthesis of inflammatory cytokines, impaired retrograde transport of neurotrophins, age-related change to connective tissue biomechanics and increased vascular permeability^25^,^41^,^42^,^43^,^44^,^45^,^46^.

Interruption of the blood supply to an organ has a wide variety of metabolic consequences, and the process of reperfusion *per se* injures cells further, being accompanied by generation of free radicals and the synthesis of inflammatory cytokines^45^,^47^,^48^,^49^. We found that patients in the fourth SBP SD or CV quartiles were significantly more likely to develop POAG than were those of the other quartiles (HRs, 1.256; 95% CI, 1.030–1.531; 1.238; 95% CI, 1.016–1.508, respectively). Such BP variability may cause ischemia-reperfusion injury of retinal ganglion cells, triggering the development of clinical POAG. Our data are very important, because the association between POAG development and SBP variability remained statistically significant after adjustment for both SBP and DBP. Hypertension is also significantly associated with POAG development. However, subjects with the same mean SBP level, who exhibit large visit-to-visit SBP variability, are at greater risk of POAG development. The SBP variability was more significant in this context than was the DBP variability or the difference between SBP and DBP.

Our Kaplan-Meier survival curves showed that patients in Q4 of SBP variability developed POAG more frequently than did those in the other three quartiles. The SBP variability in the Q4 group was 19.0 ± 6.2 mmHg; the lowest variation was 13.4 mmHg. This suggests that a useful predictive cut-off in SBP variability would be about 13 mmHg. If the SBP variability is higher than this figure, close monitoring is required in terms of POAG development.

A strength of our study is that the KNHIS, which contains data from a longitudinal cohort, is a large population-based database and, thus, an excellent resource. It was possible to track medical records and evaluate the risk of POAG in terms of BP variability. However, several limitations of our study are apparent. First, the diagnoses of POAG and other comorbidities were based on ICD-10 codes; these diagnoses may be less accurate than those obtained by medical chart review. However, the KNHIS data have been validated by reference to the rates of prevalence of 20 major diseases; the rates of prevalence were similar. Second, the KNHIS is a medical claim-based database, and POAG may have been underdiagnosed because many POAG patients are asymptomatic in the early stages of disease. Third, the KNHIS-NSC database does not include any specific code for NTG, which accounts for 77% of all POAG cases in Korean patients^50^. Thus, we cannot conclude that our findings are applicable to all POAG subtypes. In addition, we studied Koreans only; our results cannot be automatically generalized to other ethnic populations. Fourth, ambulatory BP monitoring (which was not performed) would have been optimal. However, this test modality is not always available and is often poorly tolerated, being thus inappropriate for routine assessment of long-term BP variability in clinical practice^23^. We performed sensitivity analyses on selected subjects and additional Cox’s proportional hazard regression analysis in subjects with BP measurements more than 3 times, and confirmed that data from the study population did not differ from those of subjects for whom BP was measured more than three times (S1. Table). Fifth, we could not control the types of BP medication prescribed and there was no information on the duration of systemic hypertension. Difference types of BP medications might affect differently on BP fluctuation. In terms of preferential reduction of the difference between SBP and DBP, ACE-inhibitors, diuretics, dihydropyridine calcium antagonists and vasopeptidase inhibitors seem to be more effective than beta-blockers^51^ We performed additional analysis to exclude the effect of BP medication, and multivariate Cox’s regression analysis showed that subjects without BP medication in the fourth quartiles of SBP SD or CV were significantly more likely to develop POAG than subjects without BP medication in the first to third quartiles of SBP SD or CV (S2. Table). Further studies including subgroup analysis according to the types of BP medications and information on the duration of systemic hypertension are needed to confirm our data, and to clarify the pathophysiological mechanism responsible for the association between POAG and BP variability. Finally, this study was based on the KNHIS database which didn’t contain ocular examinations including IOP. Although we focused on the systemic risk factor, it would be better to obtain information about ocular perfusion pressure also.

In conclusion, our present population-based, retrospective, longitudinal cohort study showed that SBP variability was a significant predictor of POAG development, both before and after adjustment for possible confounding factors. Patients exhibiting high visit-to-visit BP variability should be carefully monitored in terms of POAG development.

## Additional Information

**How to cite this article:** Lee, N. Y. *et al*. Fluctuation in systolic blood pressure is a major systemic risk factor for development of primary open-angle glaucoma. *Sci. Rep.* **7**, 43734; doi: 10.1038/srep43734 (2017).

**Publisher's note:** Springer Nature remains neutral with regard to jurisdictional claims in published maps and institutional affiliations.

## Supplementary Material

Supplementary Tables

## Figures and Tables

**Table 1:** Baseline characteristics of the study population comparison group (n = 79111) and primary open angle glaucoma (POAG) group (n = 910).

	Comparison group	POAG group	P-value	Age adjusted HR
	No. (%)	No. (%)		
Age (≥65 yrs)	11375 (14.38)	290 (31.87)	<0.0001	2.763 (2.403,3.177)
Sex (male)	46791 (59.15)	534 (58.68)	0.7768	1.124 (0.984,1.284)
Smoking status			0.0141	
never	50046 (71.93)	593 (76.22)		1
former	4074 (5.86)	46 (5.91)		1.142 (0.845,1.543)
current	15452 (22.21)	139 (17.87)		0.941 (0.781,1.135)
Alcohol consumption			0.0365	
none	42312 (55.3)	520 (59.43)		1
≤three times/month	12697 (16.6)	124 (14.17)		1.063 (0.871,1.298)
≥once/week	21501 (28.1)	231 (26.4)		1.093 (0.934,1.28)
Physical activity			0.0126	
no activity	37970 (49.29)	448 (51.14)		1
≤four times/week	32152 (41.74)	330 (37.67)		1.087 (0.939,1.257)
≥five times/week	6913 (8.97)	98 (11.19)		1.112 (0.893,1.383)
Diabetes mellitus (yes)	4837 (6.11)	118 (12.97)	<0.0001	1.668 (1.370,2.030)
Hypertension (yes)	15922 (20.13)	290 (31.87)	<0.0001	1.274 (1.099,1.477)
Dyslipidemia (yes)	6341 (8.02)	115 (12.64)	<0.0001	1.297 (1.065,1.580)
Household income (lowest 20%)	10536 (13.32)	127 (13.96)	0.5733	0.909 (0.753,1.098)
Residential area (urban)	35133 (44.41)	405 (44.51)	0.9539	0.900 (0.789,1.027)
Weight	63.5 ± 10.4	62.9 ± 10.3	0.0787	1.007 (1,1.1015)
Height	162.7 ± 8.8	161.2 ± 9.3	<0.0001	0.996 (0.984,1.008)
SBP	125.2 ± 16.2	128.7 ± 16.9	<0.0001	1.004 (1,1.008)
DBP	78.3 ± 10.5	79.3 ± 10.7	0.0029	1.004 (0.998,1.010)
SBP_SD	9.9 ± 6.8	11.2 ± 7.5	<0.0001	1.010 (1.001,1.018)
DBP_SD	7.0 ± 4.7	7.7 ± 5.3	<0.0001	1.015 (1.003,1.028)
DIFF_SD	7.4 ± 5.2	8.0 ± 5.8	0.0003	1.005 (0.993,1.016)
SBP_CV	7.9 ± 5.1	8.6 ± 5.6	<0.0001	1.012 (1.001,1.024)
DBP_CV	8.9 ± 5.9	9.6 ± 6.4	0.0394	1.011 (1.001,1.021)
DIFF_CV	15.7 ± 10.4	16.1 ± 11.1	0.8591	1 (0.995,1.006)

HR = hazard ratio, SBP = systolic blood pressure, DBP = diastolic blood pressure, DIFF = difference between SBP and DBP, SD = standard deviation, CV = coefficient of variation.

**Table 2:** Cox’s proportional hazard regression analysis by the quartiles (Qs) of the standard deviations (SDs) or coefficient of variations (CVs) of systolic blood pressure (SBP), diastolic blood pressure (DBP), and the blood pressure difference (DIFF).

SDs	EVENT	YEAR	PERSON YEAR*	Model 1	Model 2	Model 3	Model 4
SBP							
Q1	198	140678.5	1.41	1	1	1	1
Q2	198	138009.62	1.43	1.027(0.843,1.251)	0.992(0.799,1.232)	0.992(0.799,1.232)	0.995(0.801,1.235)
Q3	207	138576.3	1.49	1.092(0.898,1.328)	1.065(0.86,1.319)	1.055(0.851,1.307)	1.052(0.849,1.303)
Q4	307	139867.44	2.19	1.271(1.061,1.523)	1.307(1.075,1.589)	1.261(1.036,1.536)	1.256(1.03,1.531)
DBP							
Q1	210	136205.83	1.54	1	1	1	1
Q2	194	142471.35	1.36	0.972(0.799,1.182)	0.999(0.804,1.241)	1.002(0.807,1.244)	1(0.805,1.242)
Q3	242	137912.03	1.75	1.178(0.978,1.417)	1.253(1.023,1.535)	1.244(1.015,1.524)	1.245(1.016,1.526)
Q4	264	140500.64	1.88	1.129(0.942,1.354)	1.215(0.996,1.482)	1.181(0.968,1.442)	1.179(0.966,1.44)
DIFF							
Q1	231	139742.38	1.65	1	1	1	1
Q2	196	138235.79	1.42	0.964(0.795,1.167)	0.992(0.804,1.222)	0.994(0.806,1.225)	0.992(0.805,1.223)
Q3	217	138498.1	1.57	0.966(0.802,1.163)	0.963(0.786,1.181)	0.953(0.777,1.169)	0.948(0.773,1.162)
Q4	266	140606.59	1.89	0.986(0.825,1.178)	1.014(0.836,1.231)	0.986(0.812,1.198)	0.97(0.797,1.181)
SBP							
Q1	202	137991.9	1.46	1	1	1	1
Q2	199	140761.09	1.41	1.002(0.823,1.219)	0.979(0.79,1.214)	0.982(0.791,1.217)	0.986(0.795,1.223)
Q3	223	139299.92	1.60	1.126(0.931,1.363)	1.143(0.928,1.406)	1.131(0.919,1.392)	1.13(0.918,1.391)
Q4	286	139078.94	2.06	1.232(1.028,1.477)	1.266(1.04,1.541)	1.233(1.012,1.502)	1.238(1.016,1.508)
DBP							
Q1	220	139294.43	1.58	1	1	1	1
Q2	203	137615.15	1.48	1.019(0.841,1.234)	1.061(0.859,1.31)	1.062(0.86,1.311)	1.059(0.857,1.307)
Q3	227	140946.21	1.61	1.066(0.885,1.283)	1.093(0.891,1.342)	1.088(0.887,1.335)	1.093(0.89,1.341)
Q4	260	139234.07	1.87	1.12(0.935,1.34)	1.22(1.003,1.483)	1.194(0.982,1.453)	1.199(0.985,1.459)
DIFF							
Q1	242	139263.81	1.74	1	1	1	1
Q2	209	139244.6	1.50	0.932(0.774,1.122)	0.969(0.792,1.186)	0.966(0.789,1.182)	0.962(0.786,1.178)
Q3	215	139357.54	1.54	0.945(0.786,1.136)	0.921(0.752,1.129)	0.918(0.75,1.125)	0.921(0.752,1.129)
Q4	244	139216.91	1.75	0.954(0.798,1.140)	0.997(0.821,1.21)	0.983(0.81,1.193)	0.981(0.808,1.191)

^*^Incidence per 1000 persons

Model 1: adjusted for age and sex. Model 2: adjusted for age, sex, smoking status, alcohol consumption, physical activity, and household income. Model 3: adjusted for age, sex, smoking status, alcohol consumption, physical activity, and household income, diabetes mellitus and dyslipidemia. Model 4: adjusted for age, sex, smoking status, alcohol consumption, physical activity, and household income, diabetes mellitus, dyslipidemia SBP and DBP.

**Table 3:** Comparison of characteristics of the study population by SBP SD quartiles (Qs).

Group (No)	Q1–Q3 (59900)	Q4 (20121)	P-value
	No. (%)	No. (%)	
Age (≥65 yrs)	7030 (11.74)	4635 (23.04)	<0.0001
Sex (male)	36176 (60.39)	11149 (55.41)	<0.0001
Smoking status			<0.0001
never	37336 (70.99)	13303 (74.91)	
former	3238 (6.16)	882 (4.97)	
current	12017 (22.85)	3574 (20.13)	
Alcohol consumption			<0.0001
none	31396 (54.15)	11436 (58.94)	
≤three times/month	10103 (17.42)	2718 (14.01)	
≥once/week	16482 (28.43)	5250 (27.06)	
Physical activity			<0.0001
no activity	28038 (48.00)	10380 (53.22)	
≤four times/week	25266 (43.26)	7216 (37.00)	
≥five times/week	5103 (8.74)	1908 (9.78)	
Diabetes mellitus (yes)	3185 (5.32)	1770 (8.80)	<0.0001
Hypertension (yes)	9381 (15.66)	6831 (33.95)	<0.0001
Dyslipidemia (yes)	4273 (7.13)	2183 (10.85)	<0.0001
Household income (lowest 20%)	7447 (12.43)	3216 (15.98)	<0.0001
Residential area (urban)	32678 (54.55)	11805 (58.67)	<0.0001
SBP	123.7 ± 14.2	130 ± 20.4	<0.0001
DBP	77.5 ± 9.8	80.5 ± 12.1	<0.0001
SBP_SD	6.9 ± 3.5	19.0 ± 6.2	<0.0001
DBP_SD	6.0 ± 3.8	10.2 ± 5.8	<0.0001
DIFF_SD	6.1 ± 4.0	11.0 ± 6.4	<0.0001
SBP_CV	5.6 ± 2.9	14.6 ± 4.4	<0.0001
DBP_CV	7.7 ± 4.9	12.5 ± 7.0	<0.0001
DIFF_CV	13.5 ± 8.8	22.2 ± 12.1	<0.0001

SBP = systolic blood pressure, DBP = diastolic blood pressure, DIFF = difference between SBP and DBP, SD = standard deviation, CV = coefficient of variation.
