# Supplementary material for: Fluctuation in systolic blood pressure is a major systemic risk factor for development of primary open-angle glaucoma
Source: Sci Rep. 2017 Mar 6;7:43734. doi: 10.1038/srep43734 (PMC5338023; doi:10.1038/srep43734)
Supplement: Supplementary Tables [file srep43734-s1.pdf]

# Fluctuation in systolic blood pressure is a major systemic risk factor for development of primary open-angle glaucoma

Na Young Lee, M.D., Ph.D[1], Younhea Jung, M.D.[2],

Kyungdo Han[3], Chan Kee Park, M.D., Ph.D[2]

Department of Ophthalmology and Visual Science, Incheon St. Mary's Hospital, College of Medicine, The Catholic University of Korea, Seoul, Korea[1]

Department of Ophthalmology and Visual Science, Seoul St. Mary's Hospital, College of Medicine, The Catholic University of Korea, Seoul, Korea[2]

Department of Biostatistics, The Catholic University of Korea, Seoul, Korea[3]

**S 1. Table.** Cox's proportional hazard regression analysis in subjects with BP measurements more than 3 times.

| | EVENT | PERSON YEAR | IR (per 1,000) | Model1 | Model2 | Model3 | Model4 |
|---|---|---|---|---|---|---|---|
| SBP_QQ | | | | | | | |
| Q1-3 | 347 | 269281.7 | 1.289 | 1 | 1 | 1 | 1 |
| Q4 | 161 | 75742.1 | 2.126 | 1.313(1.083,1.591) | 1.425(1.16,1.75) | 1.351(1.095,1.665) | 1.347(1.091,1.664) |
| DBP_QQ | | | | | | | |
| Q1-3 | 350 | 255333.8 | 1.371 | 1 | 1 | 1 | 1 |
| Q4 | 158 | 89690.0 | 1.762 | 1.102(0.911,1.333) | 1.144(0.932,1.403) | 1.093(0.889,1.344) | 1.092(0.888,1.343) |
| DIFF_QQ | | | | | | | |
| 0 | 356 | 255840.1 | 1.391 | 1 | 1 | 1 | 1 |
| 1 | 152 | 89183.7 | 1.704 | 0.992(0.817,1.205) | 1.031(0.837,1.271) | 0.994(0.806,1.227) | 0.988(0.798,1.222) |
| SBP_QQ2 | | | | | | | |
| Q1-3 | 352 | 264718.7 | 1.330 | 1 | 1 | 1 | 1 |
| Q4 | 156 | 80305.2 | 1.943 | 1.235(1.019,1.496) | 1.337(1.089,1.641) | 1.281(1.042,1.575) | 1.284(1.044,1.579) |
| DBP_QQ2 | | | | | | | |
| Q1-3 | 357 | 257390.9 | 1.387 | 1 | 1 | 1 | 1 |
| Q4 | 151 | 87632.9 | 1.723 | 1.104(0.911,1.338) | 1.194(0.972,1.465) | 1.16(0.944,1.425) | 1.171(0.953,1.439) |
| DIFF_QQ2 | | | | | | | |
| Q1-3 | 375 | 258460.0 | 1.451 | 1 | 1 | 1 | 1 |
| Q4 | 133 | 86563.8 | 1.536 | 0.957(0.784,1.168) | 1.002(0.81,1.241) | 0.985(0.795,1.22) | 0.985(0.795,1.22) |

**S 2. Table.** Cox's proportional hazard regression analysis in subjects without BP medication.

| SDs | EVENT | PERSON YEAR | IR (per 1,000) | Model1 | Model2 | Model3 | Model4 |
|---|---|---|---|---|---|---|---|
| SBP | | | | | | | |
| Q1-3 | 446 | 352252.3 | 1.266 | 1 | 1 | 1 | 1 |
| Q4 | 174 | 92499.1 | 1.881 | 1.222(1.022,1.461) | 1.278(1.052,1.554) | 1.270(1.045,1.544) | 1.250(1.026,1.524) |
| DBP | | | | | | | |
| Q1-3 | 467 | 346882.9 | 1.346 | 1 | 1 | 1 | 1 |
| Q4 | 153 | 97868.5 | 1.563 | 1.046(0.871,1.256) | 1.112(0.912,1.357) | 1.112(0.912,1.357) | 1.101(0.902,1.344) |
| DIFF | | | | | | | |
| Q1-3 | 471 | 345363.9 | 1.364 | 1 | 1 | 1 | 1 |
| Q4 | 149 | 99387.5 | 1.499 | 0.937(0.778,1.13) | 0.939(0.765,1.153) | 0.934(0.761,1.147) | 0.910(0.739,1.122) |
| CVs | EVENT | PERSON YEAR | IR (per 1,000) | Model1 | Model2 | Model3 | Model4 |
| SBP | | | | | | | |
| Q1-3 | 442 | 346301.1 | 1.276 | 1 | 1 | 1 | 1 |
| Q4 | 178 | 98450.3 | 1.808 | 1.238(1.038,1.476) | 1.255(1.034,1.523) | 1.251(1.031,1.518) | 1.247(1.027,1.513) |
| DBP | | | | | | | |
| Q1-3 | 458 | 342478.8 | 1.337 | 1 | 1 | 1 | 1 |
| Q4 | 162 | 102272.6 | 1.584 | 1.092(0.912,1.307) | 1.158(0.952,1.407) | 1.157(0.952,1.407) | 1.158(0.953,1.408) |
| DIFF | | | | | | | |
| Q1-3 | 470 | 339343.4 | 1.385 | 1 | 1 | 1 | 1 |
| Q4 | 150 | 105408 | 1.423 | 0.945(0.786,1.137) | 0.966(0.789,1.182) | 0.962(0.786,1.178) | 0.956(0.781,1.171) |
